# A national perspective about the current work situation at modern radiotherapy departments

**DOI:** 10.1016/j.ctro.2020.08.001

**Published:** 2020-08-11

**Authors:** Jesper Lindberg, Paul Holmström, Stefan Hallberg, Thomas Björk-Eriksson, Caroline E. Olsson

**Affiliations:** a Department of Radiation Physics, Institute of Clinical Sciences, Sahlgrenska Academy, University of Gothenburg, Gothenburg, Sweden; b Department of Medical Physics and Biomedical Engineering, Sahlgrenska University Hospital, Gothenburg, Sweden; c Regional Cancer Centre West, Western Sweden Healthcare Region, Gothenburg, Sweden; d Department of Oncology, Clinical Sciences, Sahlgrenska Academy, University of Gothenburg, Gothenburg, Sweden

**Keywords:** Radiotherapy, Workflow, Organization, Staff

## Abstract

**Background:**

The radiotherapy (RT) community faces great challenges to meet the growing cancer incidence, especially regarding workload and recruitment of personnel. Workflow-related issues affect involved professions differently since they have specific expertise and various roles in the workflow. To obtain an objective understanding of the current working situation and identify workflow bottle necks in RT, we conducted a national survey on this topic in 2018.

**Materials and Methods:**

All 17 (photon-based) RT departments in Sweden were invited to participate in the study, which targeted both managers and employees in RT. Descriptive statistics were calculated for each profession and for small, medium and large departments (2/3–4/≥5 linacs).

**Results:**

Altogether, 364 filled-in questionnaires were returned (32/332 managers/employees; 94% response rate). Managers reported a general need for more staff (all professions). Small departments reported no problems with waiting times (0/3); whereas 2/3 of medium and large departments did (medium: 5/8, large: 2/3). All professions had a positive attitude towards working in RT (mean = 86%, 0/100%=negative/positive attitude). Organizational issues were ranked highest among reoccurring events that were most frustrating/had most negative effect on the work environment. The most severe workflow-related problems were reported to originate at contouring.

**Conclusion:**

Future efforts to improve the modern RT workflow need to focus on how to make positive mechanisms at small departments useful in larger settings. Our data also reveal that strong leadership and improved routines at contouring are warranted by all RT professions to reduce frustration related to organizational issues and to increase work effectivity.

## Highlights

• RT staff generally enjoyed their work and was positive to change.
• An important key to enjoy work/perform well was willingness to cooperate.
• Organizational issues, shortage of time and lack of staff were current concerns.
• Contouring was the most critical bottleneck in the RT workflow.
• Small departments were less affected by work-related problems.

## 1 Introduction

The global cancer incidence is growing and thereby the need of radiotherapy (RT) [1]. Around 50% of European cancer patients need RT but there is a large difference between actual and optimal use of RT [2]. To optimize utility and increase patient throughput, an increase of treatments is needed. However, RT resources are limited both worldwide as well as in Sweden, and it is also challenging to recruit staff [3]. RT departments will have to streamline working methods to meet future demands but it is not obvious which parts of the RT process that are best targeted for this purpose.

Short waiting time to RT is an essential factor for tumor control, especially for patients with rapidly growing tumors [4]. Postponed treatment sessions that lead to a longer overall treatment time can also reduce chances of cure [5]. With a high RT demand, limited resources and a need for short waiting times, maintaining a good working environment is challenging for the staff but extremely important to minimize stress and dissatisfaction [6]. All healthcare professions have their own area of expertise and in RT collaborations between engineers, nurses, physicians, and physicists are important for a smooth workflow (N.B. in Sweden oncology as a specialty includes all non-surgical treatments of cancer as well as RT and nurses working in RT have similar roles as a radiation therapist and dosimetrist in other parts of the world) [7]. With different roles in the RT workflow, issues originating from the same part of the process can affect the staff differently. We need to identify the most critical bottlenecks and other important factors for workflow-related issues when searching for solutions to improve the RT experience for both patients and staff.

The aim of this work was to obtain an objective understanding of the work situation at modern RT departments. We collected information about this by study-specific questionnaires on a national scale in Sweden. Questions about the working environment, the staffś views on their work as well as on existing problems and bottlenecks in the overall RT process were also asked.

## 2 Methods and material

This study is based on information collected from RT professionals working with external beam RT in Sweden. The overall study design consisted of questionnaire development (details in supplementary material), a pilot study and a main study. The pilot study was conducted November-December 2017 at one larger and one smaller RT department with participation from seven managers and 63 employees in total. The main study was conducted February-November 2018. All photon-based RT departments in Sweden were invited to participate by e-mail. The study was approved by the Regional ethical review board in Gothenburg, Sweden (Dnr: 841-16 + T640-17).

### 2.1 Questionnaire

We developed two questionnaires, one to managers (24 questions) and one to employees (32 questions), based on a literature review, own clinical experience and interviews with healthcare professionals in RT (Supplementary material). Questions for managers focused on staffing and on how overall work at their department was organized. Managers were also asked to fill in a fact sheet (10 questions) on overall information about their department. Questions directed to employees focused on how different work tasks were done in more detail, the working environment and workflow-related problems and solutions. Answering categories primarily captured percentages of a given period of time or number of occurrences per day or week, but a continuous scale for marking positive or negative responses to statements (converted into a percentage value) as well as open-ended questions were also included.

### 2.2 Data input

One person transferred the collected information into a pre-structured data file. Another person crosschecked data input on 20 randomly selected questionnaires early in the data collection process (March 2018); this verification revealed an input error of <0.2% (4/2720 entries).

### 2.3 Statistics

For the analyses, departments were divided into three categories based on number of linear accelerators (linacs; small = 2 linacs, medium = 3–4 linacs, and large=≥5 linacs). RT tasks were divided into 16 categories (booking, mould, imaging, simulation, contouring, treatment planning, patient quality assurance (QA), machine QA, maintenance, treatment, rounds, investigations, development, education, research, and other). Employees were divided into five categories (engineers, nurses, physicians, physicists, and others). If more than one manager from the same department responded to a particular question, the departmental answer was documented according to the reply of the majority. Comparable questions between the pilot and main study were analyzed together for the final results.

Results are reported in percentages, mean (±standard deviation) or median (range), whichever most appropriate given the distribution of data. Unless otherwise stated, numbers and percentages presented below relate to answers from all responding RT departments, all responding managers or all responding employees, either in total or by profession. Comparisons between groups were done using Chi-square and Mann-Whitney U-tests. A p-value ≤ 0.05 and rate ratios (RR) with 95% confidence intervals (CIs) excluding RR = 1 where considered to indicate statistical significance. When applicable, multiple group comparisons with a same result is reported as a single value (=) but as a range or as the upper value (≤) otherwise; N/A was used to indicate mathematically/statistically undefined expressions.

Data handling and calculations were conducted in Excel, (Ver. 2016, Microsoft Corporation, Redmond, WA, USA.), MATLAB (Ver. R2018a, The MathWorks Inc., Natick, MA, USA), and SPSS (Ver. 25, International Business Machines Corporation, Armonk, NY, USA).

## 3 Results

**Fig. 1:**
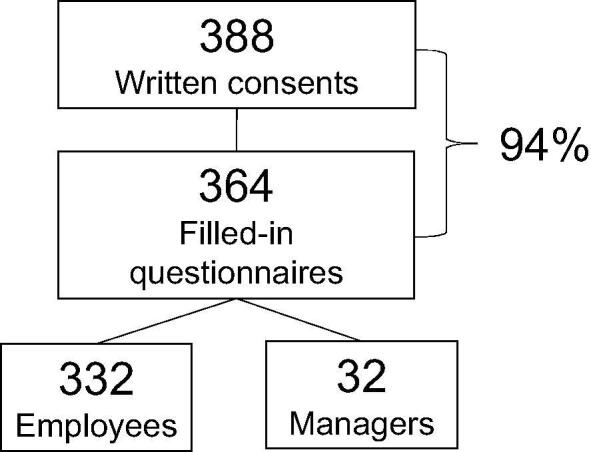
Schematic of the study recruitment procedure. Managers estimated the total number of health-care professionals working in Swedish radiotherapy to 731.

Altogether, 15/17 RT departments agreed to participate in this study, 32 managers and 332 employees of which 14 were engineers, 51 physicists, 231 nurses, 32 physicians, and 4 others (Fig. 1). All departments returned answers for the employee questionnaire, but one department did neither return answers from mangers nor on department-specific questions. RT work experience for responding managers were 7.5 ± 8.5 years and for employees 12.5 ± 10.0 years (Table 1). A majority of employees worked full-time with RT-related tasks. Among physicians, about two thirds worked part time with RT whilst having additional duties (up to full-time) elsewhere in the oncology department. Details about staffing at Swedish RT departments has recently been described [7], but in summary, per linac there are 0.4–1.3 engineers, 0.8–2.4 physicists, 1.3–8.3 nurses, 0.3–5.5 oncology nurses, 0.3–2.0 physicians and 0.3–1.5 resident physicians depending on department size. Managers reported a general need for more staff of all professions with small departments being somewhat better staffed than larger departments.

**Table 1:** How many of each profession that worked full- or part-time in radiotherapy (RT) and for how long they had been working with RT.

	Working in RT			Working years in RT			
	Full-time	Part-time		Mean + -SD	Median	Min	Max
Engineer (n = 14)	77%	23%		18.3 ± 10.2	19	1	31
Physicist (n = 51)	76%	24%		11.6 ± 8.4	10	0.5	33
Nurse (n = 231)	70%	30%		12.9 ± 11.4	10	0	43
Physician (n = 32)	38%	62%		9.1 ± 9.2	6	0.1	36
Other (n = 4*)	100%	0%		2.7 ± 1.2	2	2	4
Total (n = 332)	68%	32%		12.5 ± 10.8	10	0	43
Managers (n = 34)	n/a	n/a		7.5 ± 8.5	4	0	32

* Three responded on the question, *Working years in RT*.

Details on the use of RT equipment in Swedish healthcare have also been described previously [7]. The departments have 2–9 linacs with a majority having computer tomography (CT) located at the department (13/15 departments). All have access to magnetic resonance imaging (MRI) with large clinics having their MRI placed in-house. In total, they deliver approximately 23,500 yearly treatment series, which adds up to almost 302,000 fractions in total (16.0/13.3/12.2 fractions per series for small/medium/large departments).

### 3.1 Queuing status and waiting times to treatment

None of the small departments (0/3) reported problems with waiting times, but 2/3 of medium and large departments did, with the major reason being limited resources (medium: 5/8, large: 2/3). The major reason for patient waiting times at the seven departments without queuing problems was unforeseen events (5/7 departments). Regardless of queuing status, most common events disturbing or halting the RT-process were unexpected equipment downtime, lack of physicians or nurses, and CT-related problems (6/14 departments). Reasons for not being adequately staffed were financial (all department sizes), lack of available RT-educated staff and/or large staff turnovers (larger departments).

Twelve of the departments were working proactively to reduce the risk of waiting times, but when unexpected equipment downtime or similar events occurred immediate actions involved rescheduling of patients and of maintenance. Preventive actions in the longer perspective included scheduling of staff to meet the existing need, continuous education and planned maintenance.

### 3.2 Temporal distribution of tasks in the RT process by profession and department size

**Fig. 2:**
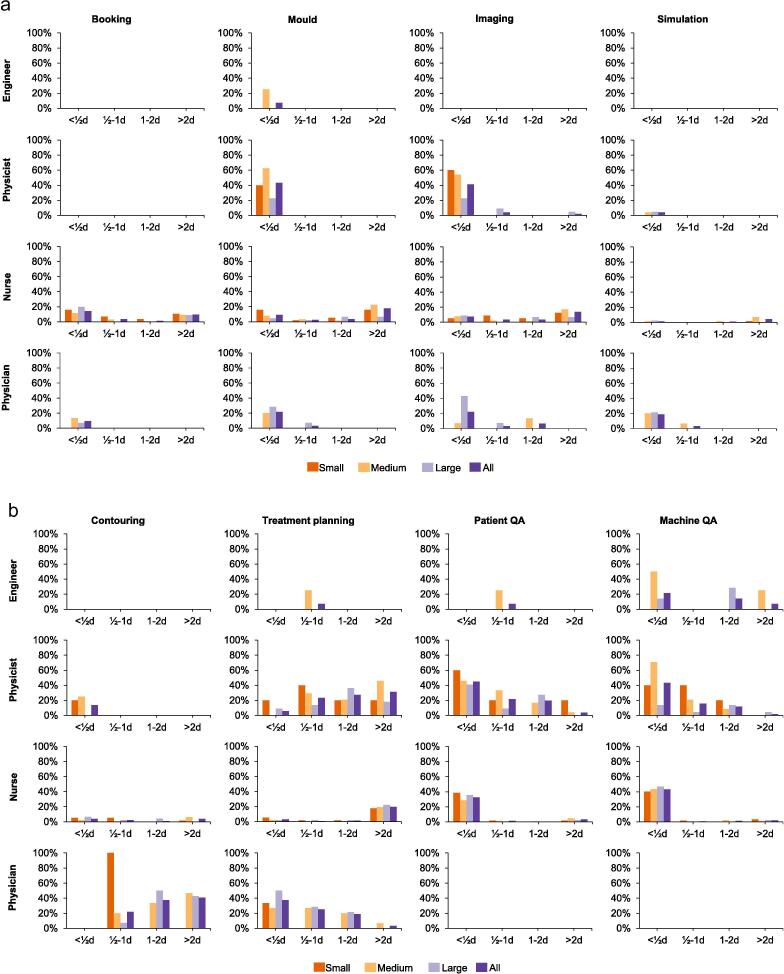

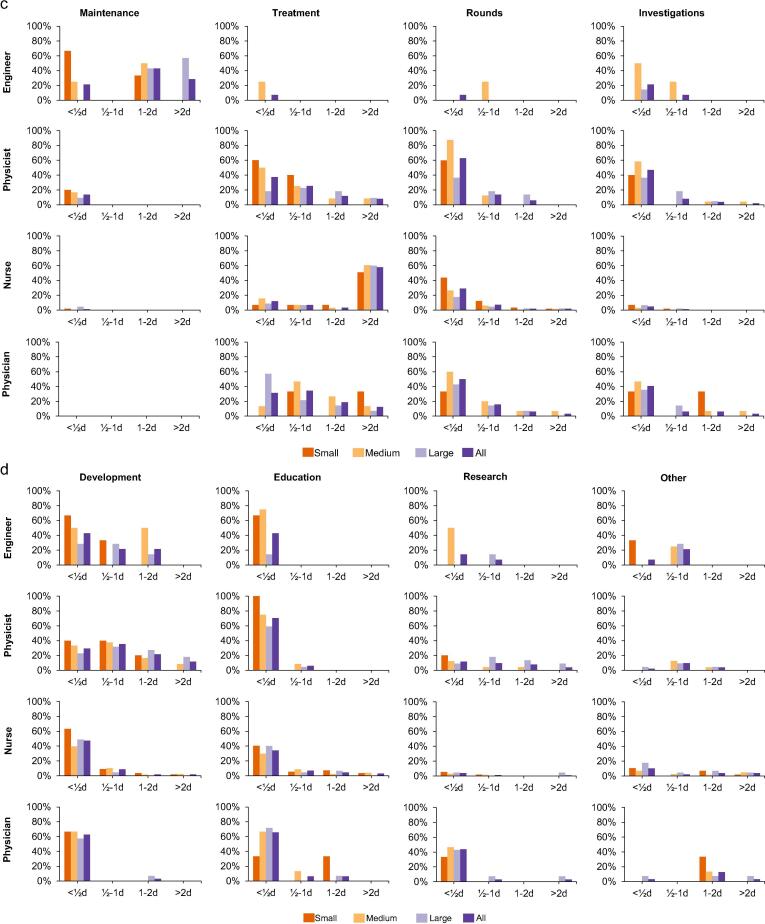
a–d: Spent time on radiotherapy tasks by profession and department size during a mean regular workweek. NB: 2/1/1 small/medium/large departments used service contracts for maintenance.

The distribution of RT tasks among personnel categories are presented in Table 2. Department size generally had small impact on how much time was spent on different tasks, in particular for nurses (Fig. 2a–d). Scheduling of treatments was the main task among those categorized as others.

**Table 2:** Description of how many from each profession that performed the listed tasks on a routinely basis, as the main performer or supporting others.

	Engineer	Physicist	Nurse	Physician	Other	Total
	(n = 14)	(n = 51)	(n = 231)	(n = 32)	(n = 4)	(n = 332)
Booking	0%	4%	35%	9%	75%	27%
Mould	0%	37%	28%	16%	25%	27%
Imaging	0%	31%	26%	13%	25%	24%
Simulation	0%	0%	5%	6%	0%	4%
Contouring	0%	24%	21%	100%	0%	28%
Treatment planning	7%	88%	25%	84%	0%	39%
Patient QA	14%	86%	48%	3%	0%	48%
Machine QA	50%	71%	48%	3%	0%	47%
Maintenance	93%	14%	2%	0%	0%	7%
Treatment	0%	82%	84%	88%	25%	80%
Rounds	7%	67%	31%	69%	0%	39%
Investigation	21%	59%	2%	47%	0%	16%
Development	71%	94%	39%	69%	0%	52%
Education	29%	71%	28%	69%	25%	39%
Research	14%	33%	4%	53%	0%	14%

Of the standard tasks, engineers primarily worked with maintenance and machine QA and spent the majority of their time doing these tasks only (Fig. 2b–c). Less time, however, was spent at smaller departments compared with larger.

Physicists worked primarily with treatment planning, patient QA and treatment, but spent little time on each of these as well as on additional tasks (Fig. 2b–c). Fewer were involved in additional tasks at larger departments but spent somewhat longer time on each of them than at the smaller departments.

Nurses worked primarily with treatment and either spent the majority of their time at this (or another) task and little time with additional tasks (Fig. 2c). More nurses at small departments spent time in rounds compared to nurses at larger departments.

All of the physicians worked with contouring and a majority with treatment and treatment planning (Fig. 2b–c). Physicians primarily spent their time at contouring and treatment, but also spent little time on many additional tasks. At small departments, less time was spent on contouring than at larger departments.

Of the non-standard tasks, activities related to development were done by every second personnel (Fig. 2d). Nurses at small departments spent more time doing development activities compared to nurses at larger departments. Research-related activities were done by less than one third of personnel except for physicians where half were involved in research. Physicists at larger departments spent more time at research compared to physicists at smaller departments.

### 3.3 Learning and support for routine RT tasks in the clinic

Tasks taking the longest time to learn were reported as contouring, maintenance and treatment planning by both managers and employees. Work instructions (in writing or as templates) existed for >75% of the routine tasks according to 78% of managers and 52% of employees. More instructions were generally available at smaller departments (small vs. medium/large: 87% vs. 81%/65% had instructions for >50% of tasks; p ≤ 0.004). There were less instructions for physicians than for the other professions (physicians vs. other professions: 9% vs. 35–62% had instructions for >75% of tasks; p ≤ 0.015; Supplementary Fig. A1). The usefulness of available written instructions and templates were on average rated as 71% and 74%, respectively (Supplementary Table A1).

### 3.4 Dependencies, disruptions and disturbances in the RT process by profession

**Fig. 3:**
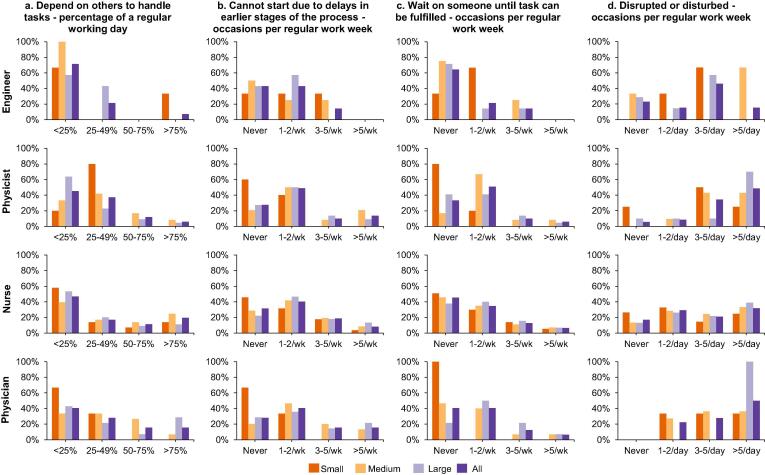
a–d: Radiotherapy workflow issues by profession and department size. (a) Dependence on others to be handled tasks; (b) Cannot start a task due to delays in earlier stages of the process; (c), Cannot complete a task because having to wait on someone; (d) Disrupted/disturbed to an extent affecting work-effectivity. (a): during a regular day; (b)-(d) during a regular week.

When asked to what extent RT staff *depended on others to hand over tasks to them*, every second respondent reported to be little or more dependent on others (Fig. 3a). Physicists and physicians at medium/large departments depended more on others than at the small departments (physicists and physicians at small vs. medium/large: 0% and 0% vs. 25/14% and 33/36%, respectively, depended on others ≥ 50% of a regular working day; p = N/A).

When asked to what extent *staff could not start their own work in time due to delays in earlier stages of the RT process*, 2/3 of the respondents reported to be affected by this regardless of profession (Fig. 3b). Physicists and physicians at the small departments were, however, rarely affected by such delays (physicists and physicians at small vs. medium/large: 0% and 0% vs. 29/23% and 33/36%, respectively, had to wait ≥3 times per week; p = N/A).

When being asked to what extent RT staff *could not complete their task due to needing to wait for input from others*, every second respondent reported to be affected by this (Fig. 3c). Only 20% of the physicists and none of the physicians had to wait for someone before they could complete a task at small departments.

Of the ten departments responding to overall disruptions or disturbances, 85% was affected by this to an extent where it had an effect on the work-efficiency per day (Fig. 3d). Engineers were the least disrupted or disturbed compared with other professions (engineers vs. other professions: 23% vs. 0–17% reported to never or rarely be disturbed; p ≥ 0.05; N/A) whilst physicists and physicians were the most disrupted or disturbed (physicists and physicians vs. other professions: 91% and 100% vs. 77–83% were disturbed at least once per day; p ≥ 0.05; N/A). Staff at medium/large departments were disrupted/disturbed to a larger degree than staff at small departments.

### 3.5 Professionals views on working in RT

The overall attitude towards working in RT was positive (86% for all professions, range 69%-93%). The lowest rate was reported by physicists working at large departments and highest by engineers working at small departments (Table 3). Small departments generally had the higher values and large departments had the lower values for all professions (small vs. large: 90% vs. 81%; p ≥ 0.05).

**Table 3:** Mean values of respective professions attitude towards working in radiotherapy, per department size (small, medium and large) and all together, rated between 0% (not enjoying) and 100% (enjoying much).

Profession	Small	Medium	Large	All
Engineer (n = 14)	93%±6	79%±10	81%±12	83%±11
Physicist (n = 51)	84%±18	81%±17	69%±33	78%±22
Nurse (n = 231)	90%±10	86%±12	84%±16	87%±12
Physician (n = 32)	83%±12	87%±10	91%±14	87%±11
Other (n = 4)				91%±8
Total	90%±10	85%±13	81%±21	86%±14

When being asked to rank the most important factor for enjoying work and performing well given sufficient staffing and equipment, all professions selected cooperation within or between professions (119/226). They also ranked organizational issues as the most frustrating recurring problem (45/228). Shortage of time and lack of staff, in particular physicians, were other concerns. Contouring was the most critical bottle neck in the RT process followed by issues originating at treatment, booking and treatment planning in that order. When problems occurred, in 213/384 of cases other actions than those taken were warranted (55%). Preventive measures to avoid similar situations in the future were not present in 233/393 of cases (59%).

When being asked about attitude towards trying new working strategies in RT, managers own position was positive (81%±19). They estimated their respective employees corresponding attitudes somewhat less positive (77%±19) whilst professionals themselves rated their attitude higher (84%±15), a statistically significant difference from the managers estimation (p = 0.02).

## 4 Discussion

Here, we investigated working methods at 15 of Sweden’s 17 RT departments in 2018 and the staff’s viewś on work-related issues. Using study-specific questionnaires and responses from 364 experienced healthcare professionals in RT, we found that professionals enjoyed their work and that their attitude towards change, if needed, was positive. We also identified strong work-related dependencies within as well as between professions. The overall trend was for small departments (2 linacs) to be less affected by work-related issues than larger departments (3–9 linacs), possibly due to being better staffed and working somewhat differently.

The current level of modernly-equipped RT departments per number of inhabitants in Sweden is above the average European standard [8], [9], however, staffing levels reflects the average European situation five to ten years ago [10]. On average, 12.8 fractions per series were delivered as compared to the predicted optimal access of 17.6 fractions per treatment series in Europe for 2012–2025 [11]. In two of three larger Swedish departments, difficulties to recruit personnel was also the reported reason for queuing problems. The reason for smaller departments faring better from this and the majority of other investigated perspectives can partly be explained by an eased workload. Smaller departments offer standard treatments of the most common tumor types resulting in a different mixture of patients compared to the patients treated at larger departments, which handle more rare tumors and non-standard treatments [7]. Another advantage for small departments is the shorter operational decision paths and closer collaborations throughout the whole RT process, which also explain why staff at smaller departments generally experienced less problems with waiting times and were less disturbed than at larger departments. On the other hand, the majority of small department were understaffed with respect to physicians, which possibly explains our finding of physicians at larger departments reporting a higher job satisfaction than physicians at smaller departments. With increased number of staff, the overall knowledge repository grows, and it may be easier to find support among colleagues, which is an identified factor for job satisfaction [12]. The larger departments in Sweden also have a larger variety of patients and access to advanced techniques [7]. Another aspect, which also needs to be kept in mind when interpreting differences between Swedish RT department sizes, is that all large and some medium-sized RT departments are located at University Hospitals in larger cities, with a close collaboration with academia. Small departments do typically not have this same close geographical connection to a university and explains why time dedicated to research activities among physicists and physicians was highest at large departments.

All professions indicated that issues at contouring resulted in the most severe problems when unforeseen events disturbed, or halted RT. Contouring was also one of three routine tasks in the RT process which was reported to take the longest time to learn. The main performer at contouring is the physician and the steps of the RT process involving them will be halted when few physicians are on duty. Physicians in Sweden are often involved in other parts of the oncology discipline, which also explains why, although working full-time, a large part of their time is spent outside RT. Based on data from a large RT department in the U.S., contouring was recently identified as a task difficult to oversee with contouring times varying significantly between both disease site and physician [13]. In combination with multiple daily disruptions and disturbances as seen in our data, conditions for physicians to concentrate at the tasks at contouring will be difficult. This results in more than inefficiency, the quality of their work may also be affected. Bikker et al. found that optimizing the physicians schedule for a large two-site department in The Netherlands had larger effects on waiting times than increased linac opening hours [14]. Work on waiting times by Proctor et al. also identified the physician as an important bottleneck for conditions in Coventry, United Kingdom [15]. One of their problems was that physicians only saw their own patients during the preparatory steps and when simulating the possibility for patients to see any physician the waiting times decreased. Although Swedish healthcare is organized differently, similar effects could be expected when physicians are specialized in certain diagnoses and only contour targets within their own diagnosis group. Regardless of hospital size and origin of RT department, contouring should be among the first tasks to be explored when aiming to increase efficiency of RT workflows.

Strengths of this study include the national approach to gain knowledge about current working methods, relevant research questions developed in close collaboration with individuals of the target population and a high response rate from staff at modern RT departments of different sizes with all relevant professions represented. The main weakness of this study, although having a high response rate from the individuals consenting to participate, is the limited number of responders from some of the professions, mainly engineers and others. This is partly due to an unknown number of that category choosing not to participate in our study and partly because of these categories as such being small in numbers. Although not achieving complete national participation from all 17 departments, we still believe that the results presented in this study provides a representative view about the current conditions to deliver RT in Sweden. It needs to be kept in mind, however, that we presented several descriptive comparisons between groups, where there were too few observations to demonstrate statistically significant differences.

The level of technological advancements between RT departments may differ globally, but the overall RT workflow is the same. Our community is constantly undergoing changes, which can challenge any well-functioning RT-department. MRI-only workflow is one immediate example, which will require a redesign of the traditional workflow with synthetic CT creation replacing CT imaging [16]. We believe that issues such as those we observed here must be resolved before introducing new tasks that alters the workflow. To streamline the clinical RT workflow in the future using automated/AI strategies, our community will have to also make more of data such as the treatment workflow metrics reported by Cardan et al. [13].

In conclusion, to improve conditions for delivering RT, actions like enabling disturbance-free contouring for physicians is critical. To reduce frustration among RT staff, a strong leadership with a plan for preventive actions for reoccurring problems can be one way to reduce organizational issues. An increased understanding of the many interruptions and dependencies different RT professions struggle with on a daily basis is another area of improvement. We suggest that future research and efforts to improve the modern RT workflow primarily focus on positive mechanisms at small departments and how to make them useful in a larger setting. One strategy to achieve short operational decision paths and to promote collaboration could be to introduce coordinators responsible for specific tasks of the RT workflow whom together organize the daily work, monitor the need for specific actions and regularly update leadership about the current status. Finally, it is important to acknowledge existing problems in the RT workflow before introducing new tasks, and potentially new problems, in the clinic.

## Declaration of Competing Interest

The authors declare that they have no known competing financial interests or personal relationships that could have appeared to influence the work reported in this paper.
