# Supplementary material for: A national perspective about the current work situation at modern radiotherapy departments
Source: Clin Transl Radiat Oncol. 2020 Aug 11;24:127–34. doi: 10.1016/j.ctro.2020.08.001 (PMC7451821; doi:10.1016/j.ctro.2020.08.001)
Supplement: Supplementary data 1 [file mmc1.docx]

**Supplementary Material**

**Questionnaire**

Two study-specific paper-based questionnaires, to managers and to employees, and one fact sheet on departmental resources and utility rates were designed. Questionnaire items and answering categories were identified based on a literature review, clinical experience and interviews with healthcare professionals of the target population.

The questionnaire for managers contained 24 questions focusing on staffing and on how overall work at their respective department was organized. The questionnaire for employees contained 32 questions and focused on how different work tasks were done in more detail, the working environment, as well as workflow-related problems and solutions. The department-specific fact sheet included ten questions on overall information about the department. Neither the manager questionnaire nor the departmental fact sheet needed to be notably changed between the pilot and the main study. The employee questionnaire, however, was substituted with three questions, two modifications and some minor revisions after suggestions from participating individuals in the pilot study.

Answering categories were primarily pre-determined, but a continuous scale for marking (*c.f.* visual analogue scale) as well as open-ended questions were included. Closed-ended questions typically had answering categories relating to percentages of a given period of time (e.g. *<25%, 25-50%, 51-75%, >75%* of a regular work-day) or number of occurrences per day/week (e.*g. Never or almost never, 1-2 times, 3-4 times or >5 times*, with *Never or almost never* analyzed together as an indicator of ‘Never’). Open-ended questions were analyzed based on categories identified as themes/key-words given the reported information. A continuous scale was presented as a straight line between two extreme values, e.g. *No, not at all* and *Yes, very much*, with respondents instructed to indicate their answer at the point of the line corresponding to their opinion. This point was then converted into a percentage value between 0-100% based on the length of the line and directed from the least agreeing to the most agreeing statement.

**Data collection**

A project member presented the project on each site and distributed study-specific paper-based questionnaires and fact sheets on departmental resources/utility rates. Written informed consent from managers and employees were collected at the same time. A local staff member acted as study coordinator and distributed the questionnaires to those not present at the meeting. The coordinator also communicated reminders and collected/posted filled-in questionnaires back to the project group. It took around two months from study presentation to receiving the last filled-in questionnaire per site.


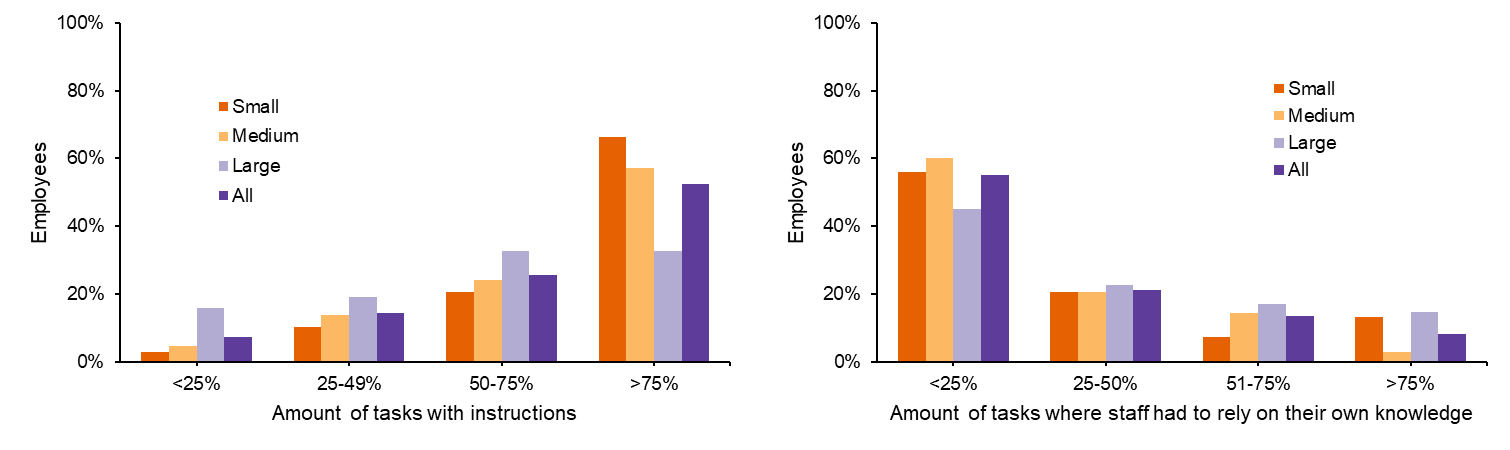


Supplementary Figure A1: Amount of tasks with written instructions (left) and amount of tasks where radiotherapy staff had to rely on their own knowledge because written instructions were missing or there were no one around to easily ask (right).

Supplementary Table A1. Mean reported usefulness of written instructions and templates when conducting new or seldom occurring tasks.

|  | Instructions | | | |  | Templates | | | |
| --- | --- | --- | --- | --- | --- | --- | --- | --- | --- |
|  | Small | Medium | Large | All |  | Small | Medium | Large | All |
| Engineer | 42% | 79% | 55% | 59% |  | 44% | 74% | 79% | 69% |
| Physicist | 59% | 81% | 67% | 73% |  | 76% | 74% | 70% | 72% |
| Nurse | 79% | 73% | 63% | 72% |  | 82% | 74% | 69% | 75% |
| Physician | 64% | 51% | 72% | 61% |  | 81% | 71% | 78% | 75% |
| Total | 75% | 72% | 65% | 71% |  | 80% | 74% | 71% | 74% |
